# The Anti-Hypercholesterolemic Effect of Low p53 Expression Protects Vascular Endothelial Function in Mice

**DOI:** 10.1371/journal.pone.0092394

**Published:** 2014-03-19

**Authors:** Francois Leblond, Steve Poirier, Carol Yu, Natacha Duquette, Gaetan Mayer, Eric Thorin

**Affiliations:** 1 Department of Pharmacology, Université de Montréal, Montreal, Quebec, Canada; 2 Department of Surgery, Université de Montréal, Montreal, Quebec, Canada; 3 Faculty of Medicine, Université de Montréal, Montreal, Quebec, Canada; 4 Research Centre, Montreal Heart Institute, Montreal, Quebec, Canada; Boston University School of Medicine, United States of America

## Abstract

**Aims:**

To demonstrate that p53 modulates endothelial function and the stress response to a high-fat western diet (WD).

**Methods and Results:**

Three-month old p53^+/+^ wild type (WT) and p53^+/−^ male mice were fed a regular or WD for 3 months. Plasma levels of total cholesterol (TC) and LDL-cholesterol were significantly elevated (p<0.05) in WD-fed WT (from 2.1±0.2 mmol/L to 3.1±0.2, and from 0.64±0.09 mmol/L to 1.25±0.11, respectively) but not in p53^+/−^ mice. The lack of cholesterol accumulation in WD-fed p53^+/−^ mice was ass–ociated with high bile acid plasma concentrations (p53^+/−^ =  4.7±0.9 *vs.* WT =  3.3±0.2 μmol/L, p<0.05) concomitant with an increased hepatic 7-alpha-hydroxylase mRNA expression. While the WD did not affect aortic endothelial relaxant function in p53^+/−^ mice (WD =  83±5 and RD =  82±4% relaxation), it increased the maximal response to acetylcholine in WT mice (WD =  87±2 *vs.* RD =  62±5% relaxation, p<0.05) to levels of p53^+/−^. In WT mice, the rise in TC associated with higher (p<0.05) plasma levels of pro-inflammatory keratinocyte-derived chemokine, and an over-activation (p<0.05) of the relaxant non-nitric oxide/non-prostacyclin endothelial pathway. It is likely that in WT mice, activations of these pathways are adaptive and contributed to maintain endothelial function, while the WD neither promoted inflammation nor affected endothelial function in p53^+/−^ mice.

**Conclusions:**

Our data demonstrate that low endogenous p53 expression prevents the rise in circulating levels of cholesterol when fed a WD. Consequently, the endothelial stress of hypercholesterolemia is absent in young p53^+/−^ mice as evidenced by the absence of endothelial adaptive pathway over-activation to minimize stress-related damage.

## Introduction

The ubiquitously-expressed tumor suppressor gene p53 is a transcription factor that is known for its capacity to regulate the cell cycle, activate DNA repair mechanisms and induce apoptosis when irreversible damage occurs [1]. Apart from its well-described roles in control of the cell cycle, repair and death, p53 has recently been linked to other key cellular functions, e.g. glucose homeostasis [2], [3], aging [4], mitochondrial respiration [5] and the regulation of oxidative stress [6]. Perturbation of any of these functions could contribute to the development of cardiovascular diseases (CVD).

p53 has been shown to be activated in advanced atherosclerotic plaque, preventing aberrant growth of vascular smooth muscle cells (VSMC) and accumulation of macrophages [7]. In addition, 24-hour p53 over-expression in rat aorta has been found to induce endothelial dysfunction [8], [9]. The deleterious effect of high p53 levels on endothelial function is likely due to decreased nitric oxide (NO) bioavailability, either by pro-oxidative p66shc up-regulation [8] or endothelium-protective Krüppel-like factor 2 reduction [9] that could result in *redox* disequilibrium. Indeed, low physiological p53 levels could up-regulate antioxidant gene expression, in contrast to high p53 levels [10]. Therefore, a model has been proposed in which p53 acts as “a guardian of the genome” under low physiological stress and promotes cell death when stress becomes irreversible [11]. We reported previously that p53 is elevated in senescent endothelial cells (EC) isolated from patients with severe coronary artery disease [12] and further increased in cells from active smokers characterized by high oxidative stress [13]. p53 expression can, however, be reduced by antioxidant treatment that delay senescence [14].

It is known that the vascular endothelium is the primary target of damage associated with CVD risk factors such as hypercholesterolemia [15]. Excess lipids increases the expression of adhesion molecules and EC permeability, lowers relaxant functions and promotes plaque development with age [16]. The role of p53 in hypercholesterolemia-induced vascular injury remains nonetheless unclear.

In this study, we postulated that low p53 levels prevent the vascular stress response attributed to a high-fat/high-cholesterol western diet (WD) and thus protect the endothelium. To test our hypothesis, partially deficient p53 mice (p53^+/−^) were placed on a WD for 3 months. Our data reveal that low p53 expression levels prevent blood cholesterol increment and endothelial function deterioration.

## Methods

### Ethics statement

The procedures and protocols in our study were approved by the Montreal Heart Institute Animal Ethics Committee and performed in accordance with the *Guide for the Care and Use of Experimental Animals of the Canadian Council on Animal Care* and the *Guide for the Care and Use of Laboratory Animals* of the US National Institutes of Health (NIH Publication No. 85–23, revised 1996).

### Animals

**Figure 1:**
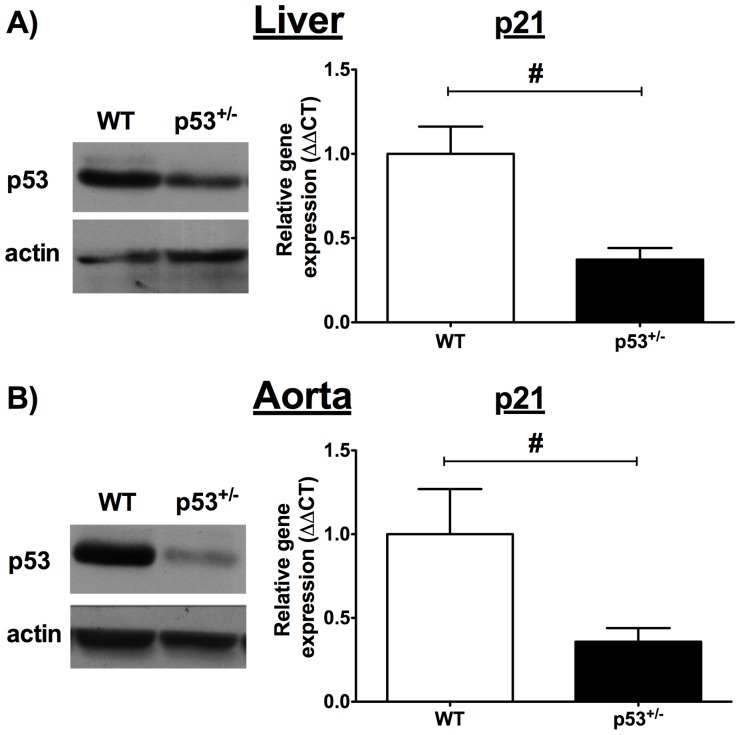
p53 protein expression and its target gene p21 mRNA expression. A) liver and B) aorta of WT and p53^+/−^ 6-month old mice. α-actin for aorta and β-actin for liver were used as internal loading controls. Data are mean±SEM ; n = 5–9. ^#^p<0.05.

Wild type C57Bl/6J (WT; B6/129PF2/J of the following parental strains: C57BL/6J-*A^w–J^* and 129P3/J (formerly 129/J)) and B6.129S2-*Trp53^tm1Tyj^*/J heterozygous (p53^+/−^) [17] 3-month-old male mice were purchased from The Jackson Laboratory (Bar Harbor, Maine, USA). B6.129S2-*Trp53^tm1Tyj^*/J mice were backcrossed at least five times to obtain a C57Bl/6J background. We confirmed low p53 protein expression and decreased mRNA expression of its target gene p21 (also known as cyclin-dependent kinase inhibitor 1A) in the liver and aorta, our tissues of interest (Fig. 1). At age 3 months, the mice were randomly selected to receive either a regular 6% fat diet (RD, 2018, Harlan Teklad Laboratories, Madison, WI, USA) or 21% fat WD (88137, Harlan Teklad Laboratories) for 3 months. See Table 1 for the main characteristics of the diet composition. Animals were kept under standard conditions (24°C; 12∶12hr light/dark cycle). During that time, blood pressure was recorded every other week using a tail-cuff device (Kent Scientific Corporation, Torrington, CT, USA). At 6 months of age, mice were fasted overnight and anesthetized (44 mg/kg ketamine and 2.2 mg/kg xylazine) the following morning. Blood was collected and plasma was frozen at –80°C. Livers were removed, snap-frozen in liquid nitrogen and kept at –80°C. Thoracic and abdominal aortas were harvested and placed in ice-cold physiological salt solution (PSS, pH 7.4, mmol/l: 119 NaCl, 4.7 KCl, 1.18 KH_2_PO_4_, 1.17 MgSO_4_, 24.9 NaHCO_3_, 1.6 CaCl_2_, 0.023 EDTA, and 10 glucose) and surrounding fat was removed. Thoracic aortas were cut in 2-mm rings for vascular reactivity studies or embedded in OCT for immunostaining. Abdominal aortas were snap-frozen in liquid nitrogen and used for western blot, enzymatic activity and qPCR studies. In order to conduct all experiments in aortas, we had to perform some of our experiments with a limited number of mice.

**Table 1:** General characteristics of the diets.

% of energy coming from	RD	WD
Carbohydrates	58.0	42.7
Fat	18.0	42.0
Protein	24.0	15.2

RD  =  regular diet, WD  =  western diet.

### Plasma parameters

Glucose and keratinocyte-derived chemokine (KC) levels were measured at the Montreal Heart Institute Clinical Biochemistry Laboratory. Total and HDL-cholesterol levels were measured independently by the Clinical Biochemistry Laboratory using the Siemens Dimension® clinical chemistry system and the Flex® reagents. LDL-cholesterol levels were then calculated with the Friedewald equation. In addition, LDL-cholesterol levels were independently measured using a commercial kit (Waco Diagnostics, Richmond, VA, USA). Insulin (Alpco Diagnostics, Salem, NH, USA), bile acids (Crystal Chem, Downers Grove, IL, USA) and the thromboxane A_2_ (TXA_2_) metabolite 11-dehydro thromboxane B_2_ (TXB_2_, (Cayman Chemical Company, Ann Arbor, MI, USA) were quantified with commercial kits according to the manufacturers’ protocols.

### Western blotting

Proteins isolated from the liver and abdominal aorta were loaded for SDS-gel electrophoresis and incubated overnight with p53 antibody (Leica Microsystems, Concord, ON, Canada). Livers and abdominal aortas were disrupted with a mortar and pestle on dry ice and kept at –80°C. Protein were extracted from tissue powder in a lysis buffer of the following composition (mM): Tris–HCl 50, β-glycerophosphate 20, NaF 20, EDTA 5, EGTA 10, Na_3_VO_4_ 1, benzamidin 10, dithiothreitol 5, PMSF 0.5, leupeptin 0.02, microcystin LR 1, and Triton X-100 1% (v/v). Thirtyμg of proteins were loaded on a 10% acrylamide SDS-PAGE gel. After 45 minutes of migration at 200 V, the gel was transferred to a nitrocellulose membrane. α-actin for aortas and β-actin for livers were used as loading controls (Sigma-Aldrich Canada, Oakville, ON, Canada).

### Reverse transcriptase-quantitative polymerase chain reaction (RT-qPCR)

Total RNA extraction was performed with RNeasy mini-kit (Qiagen Canada, Toronto, ON, Canada) following manufacturer’s protocol. Reverse transcriptase reaction (100 ng and 1 μg total RNA for aortas and liver respectively) was performed as previously [18] by using the Moloney murine leukemia virus reverse transcriptase (200 U, Invitrogen, Carlsbad, CA, USA). Primers were selected in 2 different exons spanning a large intronic sequence, to avoid amplification of genomic DNA (see Table 2 for primer sequences). Quantitative PCR (qPCR) were performed with platinum SYBR Green qPCR SuperMix-UDG (Invitrogen, Carlsbad, CA, USA). Annealing and elongation temperatures, cDNA template quantity and primer concentrations were optimized for each pair of primers to generate a standard curve with an efficacy of 100±10%. Cyclophilin A served as normalizer, and relative gene expression was calculated by the ΔΔCT method [19].

**Table 2:** Sequences of the primers for real-time quantitative PCR.

Genes	Forward (5′-3′)	Reverse (5′-3′)
Human		
Cyp7a1	AGGAACCCAGAAGCAATGAA	TCCTTAGCTGTCCGGATGTT
p21	GGAAGACCATGTGGACCTGT	TAGGGCTTCCTCTTGGAGAA
p53	CATGAGCGCTGCTCAGATAG	TGGTACAGTCAGAGCCAACCT
SHP	GCTGTCTGGAGTCCTTCTGG	ACTTCACACAGCACCCAGTG
TBP	GCTGAATATAATCCCAAGCGATTT	GCAGTTGTCCGTGGCTCTCT
Mouse		
Apoliprotein B	AAGCTGTTCAGTGGCAGCAACA	AGAGAGGCTTGCAAGTTGACCA
Cyclophilin A	CCGATGACGAGCCCTTGG	GCCGCCAGTGCCATTATG
Cyp7a1	AACGATACACTCTCCACCTTTG	CTGCTTTCATTGCTTCAGGG
FXR	TGGAGAACTCAAAATGACTCAGG	CTTTTGTAGCACATCAAGCAGG
HMG CoA reductase	AGTACATTCTGGGTATTGCTGG	ACTCGCTCTAGAAAGGTCAATC
LDLR	GTATGAGGTTCCTGTCCATC	CCTCTGTGGTCTTCTGGTAG
LXR	CGAGGTCATGCTTCTGGAG	CTCTGGAGAACTCAAAGATGGG
p21	TGTCGCTGTCTTGCACTCT	AGACCAATCTGCGCTTGGA
PCSK9	TGCAAAATCAAGGAGCATGGG	CAGGGAGCACATTGCATCC
SHP	GTCCCAAGGAGTATGCGTAC	CAGGGCTCCAAGACTTCAC
TXS	TTGGAACTCCGAGAGCGATA	CACTGTCTGCTACCATCTTG

### HepG2 cell culture experiments

HepG2 cells were seeded in 6-well plates at a density of 4×10^5^/ml. Twenty-four hours after plating, cells were incubated with either 0.1% DMSO or 0.5 μg/ml doxorubicin (DOX) in Dulbecco's Modified Eagle Medium supplemented with 10% FBS. Total RNA was extracted using RiboZol (Amresco, Solon, OH, USA) for which integrity was verified by agarose gel electrophoresis. Afterwards, cDNA was prepared from 1 μg of total RNA using the SuperScript II Reverse transcriptase (Invitrogen, Carlsbad, CA, USA) according to the manufacturer’s instructions. qPCR was performed from 1:10 dilution of the converted cDNA mixed with PerfeCTa SYBR Green SuperMix, UNG, Low ROX (Quanta Biosciences, Gaithersburg, MD, USA) using the MX3000p real-time thermal cycler (Agilent, Mississauga, On, Canada). For each gene of interest, dissociation curves and agarose gel electrophoresis were performed to ensure unique PCR product. Arbitrary unit was determined from PCR duplicates for each sample using the TATA box binding protein (TBP) as a normalizer. Oligonucleotides sequences used are listed in Table 2. Error bars are representative of four independent experiments analyzed in duplicate.

### Myograph studies

Two-mm rings were isolated from thoracic aorta and mounted on 20-μm tungsten wires in microvessel myographs (IMF, University of Vermont, Burlington, VT, USA), as described previously [20]. Vessels were stretched to obtain an optimal basal tension of 1.5 g and integrity of the vessels was tested with a 40 mM KCl-PSS solution. After 2 washout periods, the vessels were equilibrated for 30–45 minutes alone, in presence of N^ω^-nitro-L-arginine (L-NNA, 100 μM, a NOS inhibitor), indomethacin (10 μM, an non-selective cyclooxygenase inhibitor), furegrelate (10 μM, thromboxane synthase inhibitor), peg-catalase (cell permeable form of catalase, 100 U/mL) or with both L-NNA and indomethacin. The thromboxane A_2_ analog 9,11-Dideoxy-11α,9α-epoxymethanoprostaglandin F_2α_ (U46619) at a concentration of 100 nM was used to obtain a pre-constriction representing 50±10% of the maximal constriction. Endothelium-dependent and independent relaxation dose-response curves were performed using respectively acetylcholine (ACh, 0.1 nM to 30 μM) and sodium nitroprusside (SNP, 0.1 nM to 30 μM). Vessels were used for 2 consecutive dose-response curves separated by 3 washes period with fresh PSS and another incubation period of 30-45 minutes with drugs. Preliminary studies showed no effect of consecutive dose-response curves in aortas (data not included). The maximal contraction was obtained by adding 127 mM KCl-PSS on each vessel at the end of the protocols. Relaxation values were obtained for each doses with this equation: Relaxation (%)  =  100% - contraction (%).

### Superoxide dismutase 2 (SOD2) activity

SOD2 activity was assessed in abdominal aorta, as recommended in the manufacturer’s protocol (Cayman Chemical Company). It was measured in the presence of 1 mM of potassium cyanide to block copper-zinc SOD activity [21]. Values (units) were adjusted with protein content.

### Quantification of oxidative stress in aorta

The fluorescent oxidative probe dihydroethidium (DHE) (Sigma-Aldrich Canada Ltd., Oakville, ON, Canada) was used to evaluate the impact of oxidative stress on aorta sections [22], [23]. DHE is a cell permeable dye that is oxidized by O2^. −^ to ethidium bromide, which subsequently intercalates with DNA and is trapped within cell nuclei. DNA counterstaining was performed using To-Pro-3 (Molecular Probe, Burlington, ON, Canada). Frozen aortic segments from the thoracic aorta were cut into 14-μm thick sections. Sections were double-stained with a mixture of 5 μmol/L DHE and 2 μmol/L To-Pro-3. DHE fluorescence was visualized with a confocal microscope (Zeiss LSM 510, Carl Zeiss, Oberkochen, Germany; objective Plan-Apochromat, 40x/1.3, oil). DHE was excited with a HeNe laser (543 nm) and emitted light was collected between 565 and 615 nm. The camera’s acquisition settings were identical for all images. Computer-based analysis was performed using Image J software (National Institute of Health, USA) and calculated by the following equation: I =  Σ I/A, where I is the fluorescence intensity, Σ I the summation of all nuclei intensity, A the total area of the nuclei.

### Statistical analysis

*n* refers to the number of animals studied in each protocol. The results are presented as means±SEM. Half-maximum effective concentration (EC_50_) and *p*D_2_ values, negative logs of EC_50_, were measured from individual concentration-response curves. Unpaired student’s *t*-test or 2-way analysis of variance was followed by unpaired student’s *t*-test. P<0.05 values were considered to be statistically significant.

## Results

### Physiological and plasma changes

Body weight increased after 3 months on WD in both p53^+/−^ and WT mice (Table 3). WD elevated glycaemia in both strains. Blood pressure remained stable during the 3-month treatment period (Table 3). Levels of triglycerides were similar following the WD in both groups and tended to decrease (Table 3). In contrast, total cholesterol (TC), HDL-cholesterol and estimated LDL-cholesterol levels remained unchanged in p53^+/−^ mice fed WD but rose in WT mice (Table 3). However, the rise in LDL-cholesterol observed in the WT mice was not confirmed when it was directly measured suggesting the importance of non-LDL-cholesterol, i.e. the VLDL-cholesterol (Table 3). Finally, plasma levels of KC, the pro-inflammatory mouse ortholog of human interleukin-8 (IL-8), increased significantly in WT mice only (Table 3). Therefore, neither cholesterol nor KC circulating levels changed in p53^+/−^ mice when fed a WD.

**Table 3:** Body weight, SBP and plasma profile in WT and p53^+/−^ mice fed a RD or WD.

Parameters	WT RD	WT WD	p53^+/−^ RD	p53^+/−^ WD
Body weight (g)	25.8±0.5 (16)	33.0±1.6* (8)	28.1±0.8^#^ (12)	34.6±1.6* (8)
SBP (mm Hg)	162±2 (8)	161±4 (8)	157±4 (8)	158±7 (8)
Glucose (mmol/L)	14.8±1.4 (7)	20.5±2.3* (8)	14.4±2.1 (7)	20.3±3.1* (7)
Triglycerides (mmol/L)	0.53±0.07 (8)	0.33±0.04* (8)	0.50±0.08 (7)	0.39±0.08 (7)
Total cholesterol (mmol/L)	2.1±0.2 (8)	3.1±0.2* (8)	2.3±0.1 (7)	2.6±0.2 (7)
HDL (mmol/L)	1.2±0.1 (8)	1.7±0.1* (8)	1.4±0.1 (7)	1.5±0.1 (7)
LDL measured (AU)	1.00±0.06 (10)	1.13±0.09 (9)	1.10±0.12 (5)	0.96±0.10 (4)
LDL calculated (mmol/L)	0.64±0.09 (8)	1.25±0.11* (8)	0.75±0.03 (7)	0.76±0.18^#^ (7)
KC (mmol/L)	102.5±29.9 (6)	244.9±43.9* (6)	107.2±34.5 (5)	159.5±54.8 (5)
Bile acids (μmol/L)	3.0±0.5 (5)	3.3±0.2 (4)	4.2±0.7^#^ (4)	4.7±0.9^#^ (5)

Data are means±SEM. (n) mice. SBP  =  systolic blood pressure, RD  =  regular diet, WD  =  western diet, KC  =  keratinocyte-derived chemokine. *p<0.05 compared to RD with matching genotype; ^#^p<0.05 compared to WT mice with matching diet.

### Expression of genes involved in hepatic cholesterol metabolism

**Figure 2:**
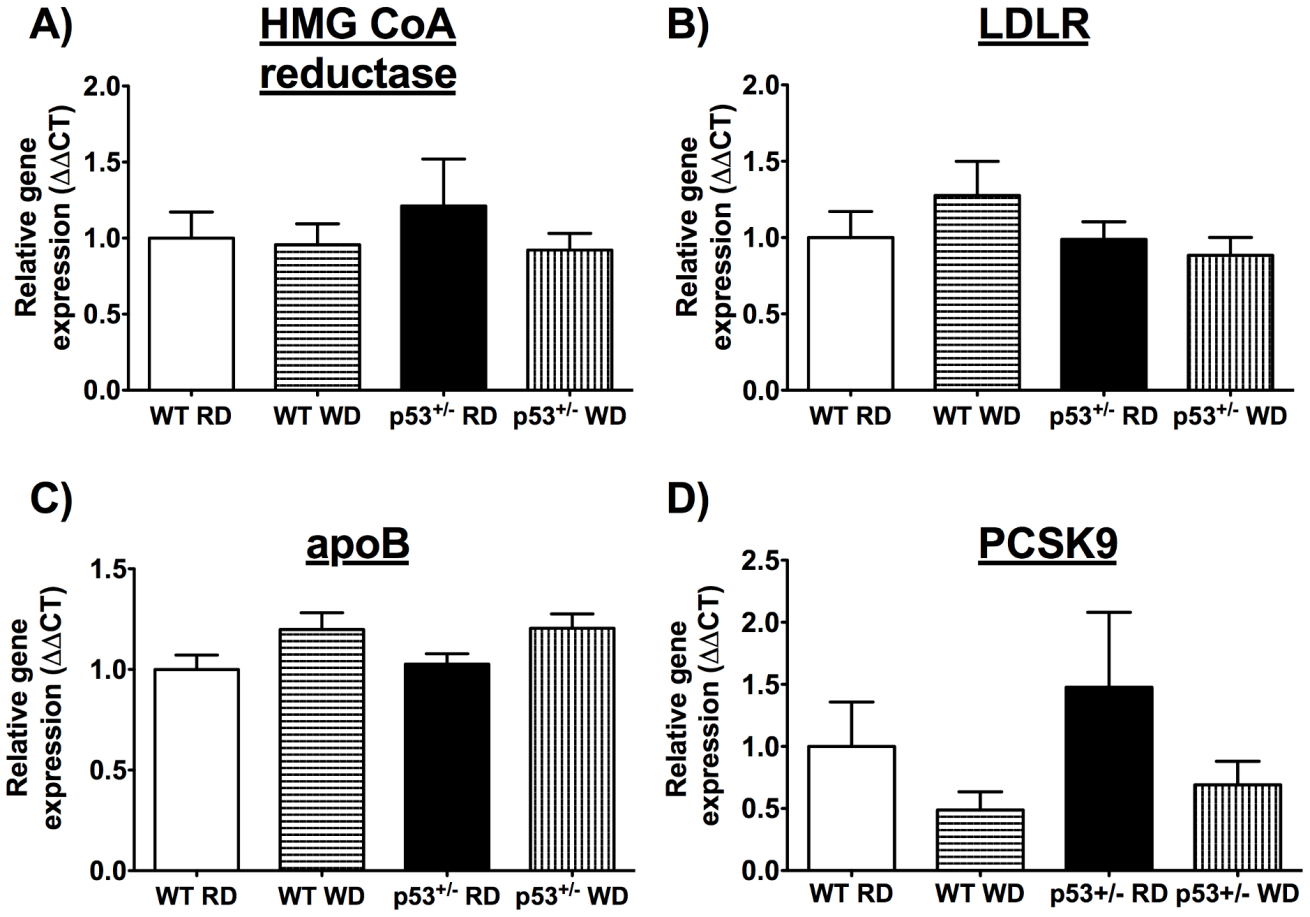
Similar synthesis and uptake of cholesterol. Hepatic mRNA expression of important genes implicated in cholesterol metabolism in WT and p53^+/−^ 6-month old mice fed a regular diet (RD) or a western diet (WD). A) HMG CoA reductase, B) LDL receptor (LDLR), C) apolipoprotein B (apoB) and D) proprotein convertase subtilisin kexin type 9 (PCSK9). Data are mean±SEM ; n = 5–7.

We quantified the expression of key genes implicated in cholesterol homeostasis in the liver: mRNA levels of the rate-limiting cholesterol synthesis enzyme HMG CoA reductase (Fig. 2A), LDL receptors (LDLR) responsible for LDL uptake (Fig. 2B), apolipoprotein B (apoB), a necessary partner for LDL in receptor binding (Fig. 2C), and proprotein convertase subtilisin kexin type 9 (PCSK9) that mediates degradation of the LDLR (Fig. 2D) were unaffected by WD in both mouse strains. Hence, the differences in circulating cholesterol levels between WT and p53^+/−^ mice on WD did not appear to originate from changes in cholesterol synthesis or liver uptake.

### Bile acid metabolism

**Figure 3:**
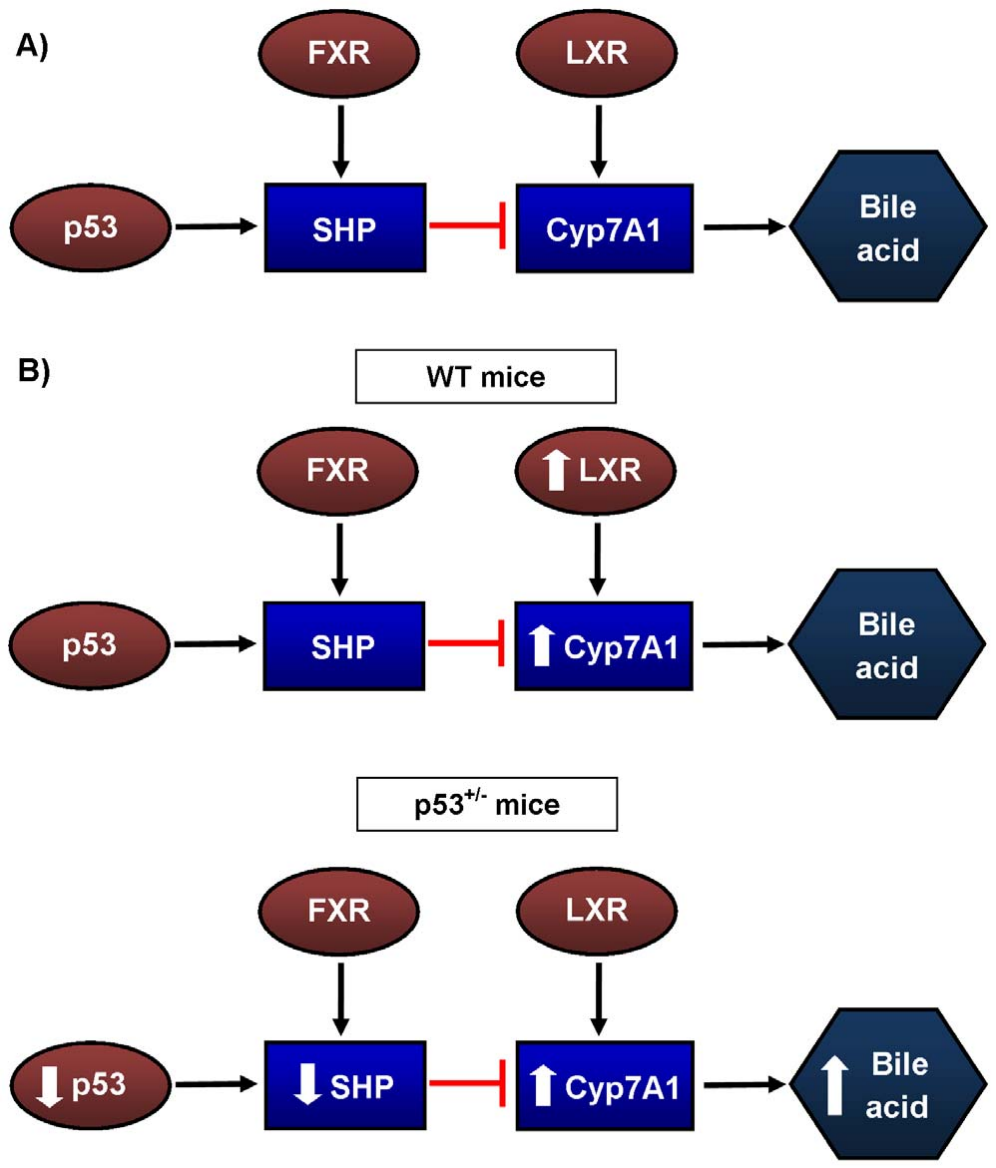
Key mediators of bile acids (BA) synthesis in the liver of mice. A) Transcription factors p53 and farnesoid X receptor (FXR) can activate the transcription of small heterodimer partner (SHP) which inhibits 7-α-hydroxylase (Cyp7A1), the first enzyme in the BA synthesis pathway. Liver X receptor (LXR) can activate transcriptionally Cyp7A1 to produce more BA. B) The Western diet (WD) activated transcription of LXR in WT mice in association with increased Cyp7A1 gene expression. Lower p53 expression in p53^+/−^ mice is associated with lower gene expression of SHP. While no difference is seen in FXR and LXR, Cyp7A1 gene expression and BA levels are elevated.

**Figure 4:**
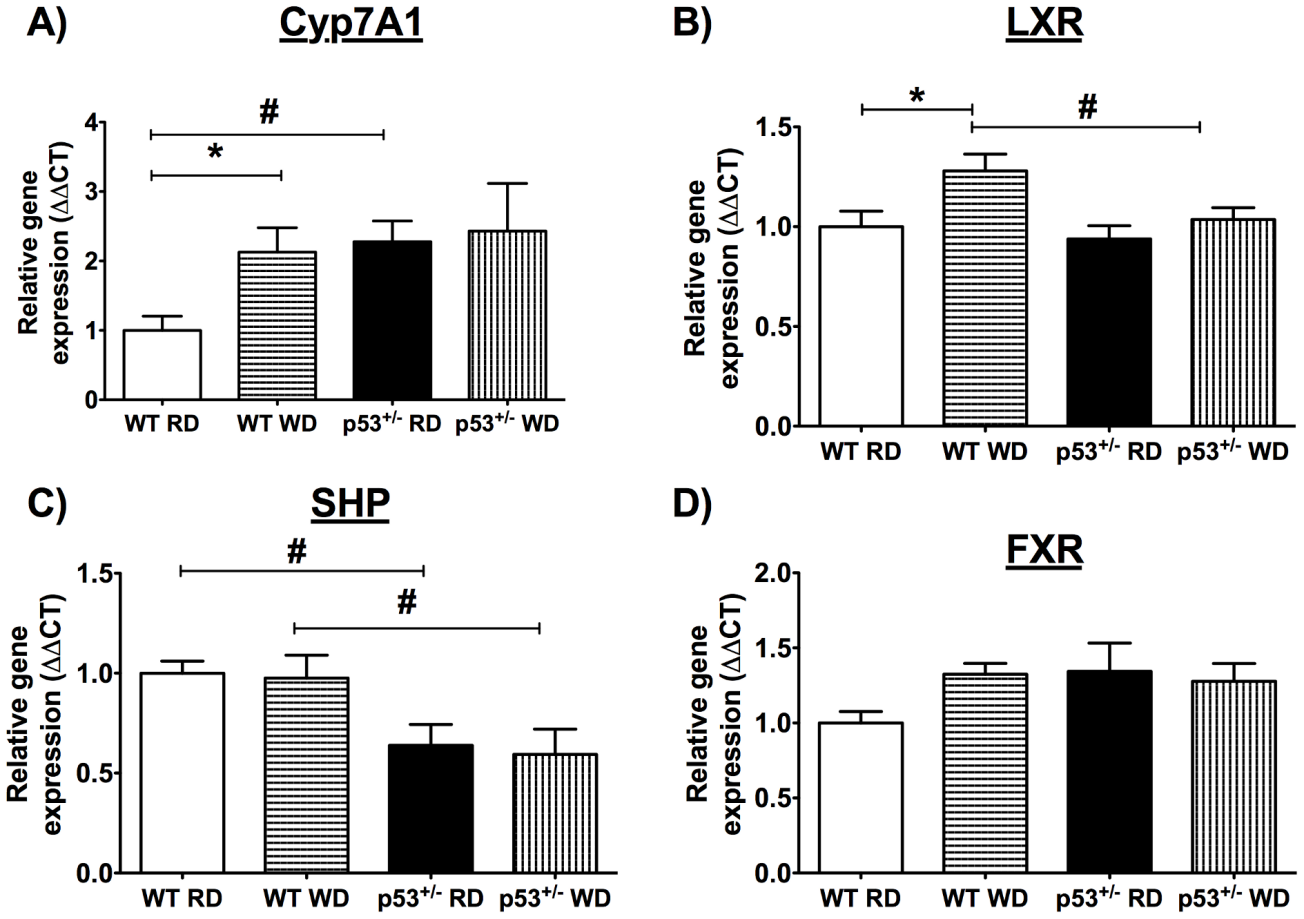
Bile acid genes expression differs between p53^+/^^−^ and WT mice. Hepatic mRNA expression of genes implicated in bile acid metabolism in WT and p53^+/−^ 6-month-old mice fed a regular diet (RD) or a 3-month Western diet (WD). A) 7-alpha-hydroxylase (Cyp7A1), B) liver X receptors (LXR), C) small heterodimer partner (SHP), and D) farnesoid X receptors (FXR). Data are means±SEM; n = 5–7. *p<0.05 compared to RD with matching genotype; ^#^p<0.05 compared to WT mice with matching diet.

Bile acid metabolism is the main cholesterol elimination pathway. In p53^+/−^ mice, circulating bile acid levels were higher with both dietary regimens compared to WT mice (Table 3). Bile acid metabolism is tightly regulated by multiple partners (Fig. 3A), and their synthesis is mainly under the control of the enzyme 7-alpha-hydroxylase (Cyp7A1) [24]. Accordingly, p53^+/−^ mice expressed more Cyp7A1 mRNA than WT mice fed RD (Fig. 4A). As expected, WD induced a 2-fold increase in Cyp7A1 gene expression in WT mice, reaching a similar expression level as in p53^+/−^ mice (Fig. 4A). Liver X receptors (LXR), which directly regulate Cyp7A1 expression [25], were augmented in WT mice on WD, while remaining unchanged in p53^+/−^ mice (Fig. 4B). Up-regulation of Cyp7A1 expression in p53^+/−^ mice is, therefore, under the control of a partner other than LXR. It has been reported that p53 directly regulates small heterodimer partner (SHP), which inhibits Cyp7A1 expression [26], [27]. SHP expression was lower in p53^+/−^ than in WT mice independently of the diet (Fig. 4C). Although SHP can be activated by farnesoid X receptors (FXR), a nuclear receptor critical in bile acid metabolism [28], FXR expression did not change significantly in either mouse strain (Fig. 4D). Altogether, these results indicate that low p53 is associated with constitutively low levels of SHP that could contribute to the up-regulation of Cyp7A1 expression and, thus, higher constitutive bile acid levels (Fig. 3B).

**Figure 5:**
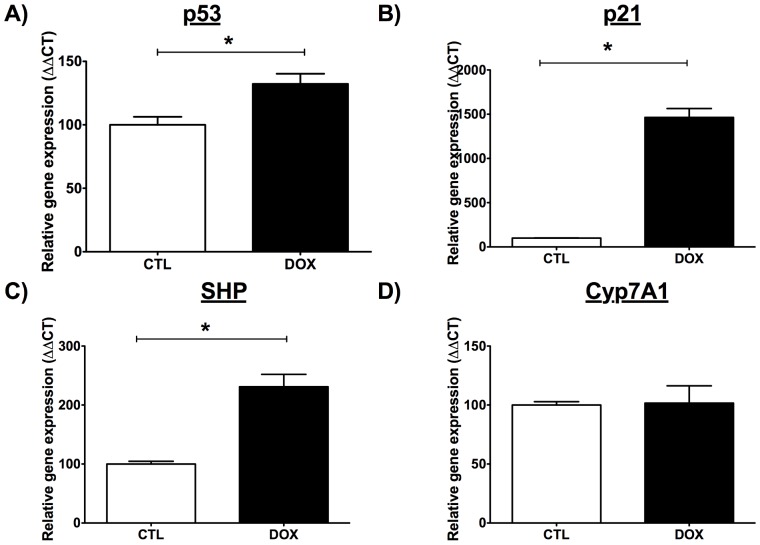
Effect of doxorubicin (DOX) on gene expression in HepG2 cells. Gene expression of A) p53, B) p21, C) SHP and D) Cyp7A1 in human hepatic HepG2 cell lines after a 24-hour incubation with 0.5 μg/ml DOX, a known-activator of p53. Data are mean±SEM of four independent experiments analyzed in duplicate.

To validate this hypothesis, we used DOX to stimulate p53 expression in HepG2 cells, a human hepatocarcinoma cell line that expresses wild-type p53. DOX, a well-known activator of p53, has previously shown to increase p53 protein and gene expressions in HepG2 cells [26], [29]. Incubation with DOX (0.5 μg/ml) for 24 hours activated genes expression of p53, p21, SHP, but not that of Cyp7A1 in HepG2 cells (Fig. 5).

### Vascular endothelial function

**Figure 6:**
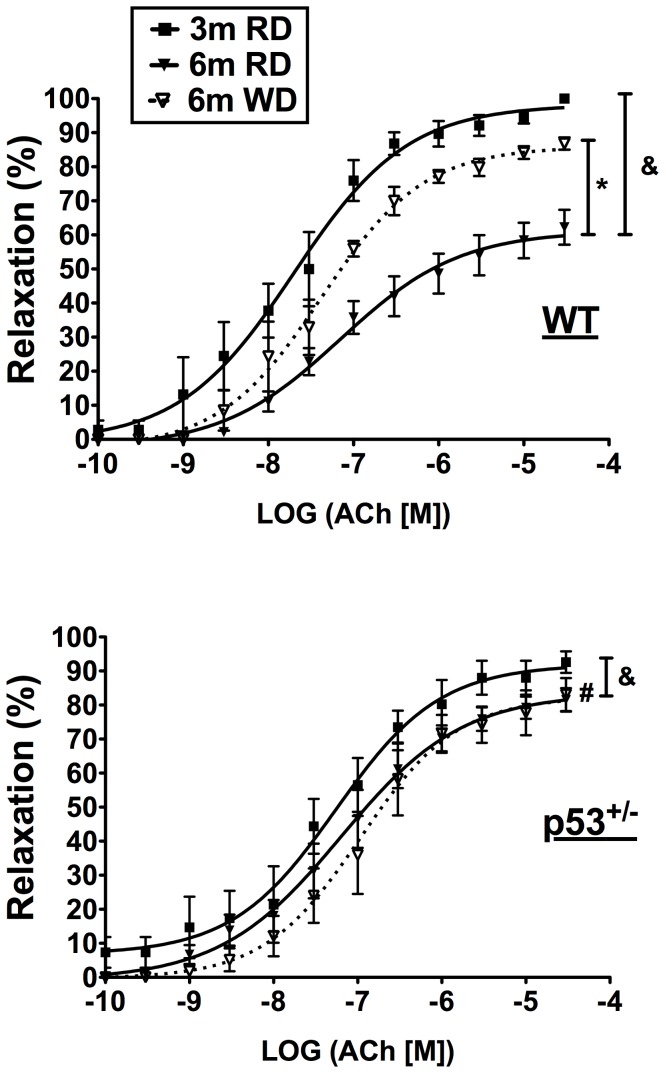
Age and WD affect differentially p53^+/^^−^ and WT mice. Aortic endothelium-dependent relaxation concentration-response curves with acetylcholine (Ach) in 3-month (3-mo) and 6-month (6-mo) WT or p53^+/−^ mice fed a regular diet (RD) or Western diet (WD). Data are means±SEM; n = 4–14. ^&^p<0.05 compared to 3-mo; *p<0.05 compared to RD; ^#^p<0.05 compared to 6-mo WT RD mice.

**Figure 7:**
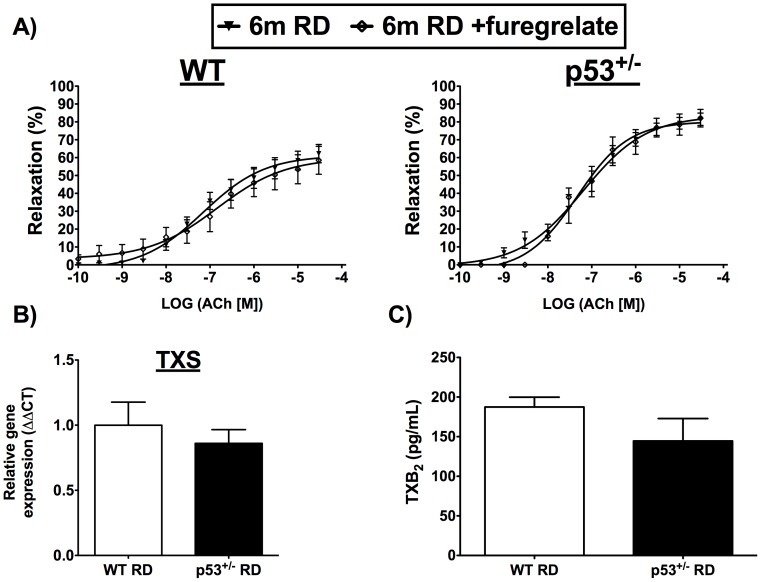
Effect at 6-month on thromboxane A_2_ pathway. Measures in WT and p53^+/−^ mice fed a regular diet (RD) of A) endothelium-dependent relaxations to acetylcholine (ACh) of aorta segments isolated from WT and p53^+/−^ mice were obtained in presence of thromboxane synthase inhibition (furegrelate, 10 μM), B) thromboxane synthase (TXS) gene expression in the aorta, C) release of the thromboxane A_2_ metabolite 11-dehydro thromboxane B_2_ (TXB_2_) in the plasma. Data are mean±SEM. n = 3–6 in (A), n = 3 in (B) and n = 4 in (C).

The vascular endothelium is highly sensitive to the metabolic and *redox* environment [30], and premature decline of its function, evidenced by decreased relaxing properties, is an early event in the presence of CVD risk factors [15]. We therefore tested the hypothesis that endothelial relaxing function is protected in p53^+/−^ mice due to the absence of elevated circulating cholesterol levels on WD. At 3 months, when fed RD, no differences in maximal relaxation (Fig. 6 and Table 4) and sensitivity (data not included) were noted between p53^+/−^ and WT mice. At 6 months, however, the maximal relaxation induced by Ach was greater in p53^+/−^ than in WT mice fed RD (Fig. 6 and Table 4), owing to a limited age-related decline in endothelial function in p53^+/−^ mice in contrast to WT mice. In WT mice on RD, in the presence of the nonspecific cyclooxygenase (COX) inhibitor indomethacin or cell-permeable PEG-catalase, maximal relaxation by Ach increased to similar values as in p53^+/−^ mice (Table 4), suggesting that the age-related decline in endothelial relaxing function in WT, but not in p53^+/−^ mice, is linked with a rise in COX1/2-associated oxidative stress. It is unlikely that this phenomenon derives from augmented TXA_2_ production, as reported previously in renal arteries [18], since the thromboxane synthase inhibitor furegrelate did not restore maximal relaxation in arterial segments isolated from WT mice (Table 4, Fig. 7A). Furthermore, gene expression of thromboxane synthase (Fig. 7B) and circulating levels of the TXA_2_ metabolite TXB_2_ were not different between the 2 mouse strains (Fig. 7C).

**Table 4:** Efficacy (E_max_) of Ach in aorta from 3-mo and 6-mo WT and p53^+/−^ mice fed RD or WD.

	WT			p53^+/−^		
	3-mo RD	6-mo RD	6-mo WD	3-mo RD	6-mo RD	6-mo WD
Ach	100.0±0 (6)	62.2±5.1^&^ (14)	86.8±1.8* (4)	92.6±3.2 (5)	81.5±3.5^&,#^ (9)	83.1±4.8 (5)
+indo	n.a	80.8±4.2^A^ (10)	80.4±6.9 (5)	n.a	85.0±5.7 (9)	74.8±8.1 (5)
+furegrelate	n.a.	58.5±7.8 (6)	n.a.	n.a.	82.1±4.9 (3)	n.a.
+PEG-catalase	n.a.	84.7±9.3^A^ (5)	85.9±2.9 (5)	n.a.	84.8±5.1 (4)	88.9±4.9 (5)
+L-NNA/indo	n.a.	36.1±4.9 (16)	57.2±3.5* (5)	n.a.	51.9±11.9 (6)	60.7±2.4 (5)

Data are means±SEM. (n) mice. Ach  =  acetylcholine, 3-mo  =  3 months of age, RD  =  regular diet, WD  =  western diet, indo  =  indomethacin. ^&^p<0.05 compared to 3-mo; *p<0.05 compared to RD with matching genotype; ^#^p<0.05 compared to WT mice with matching diet; ^A^p<0.05 compared to Ach in mice with matching genotype and diet. n.a.: not available.

**Figure 8:**
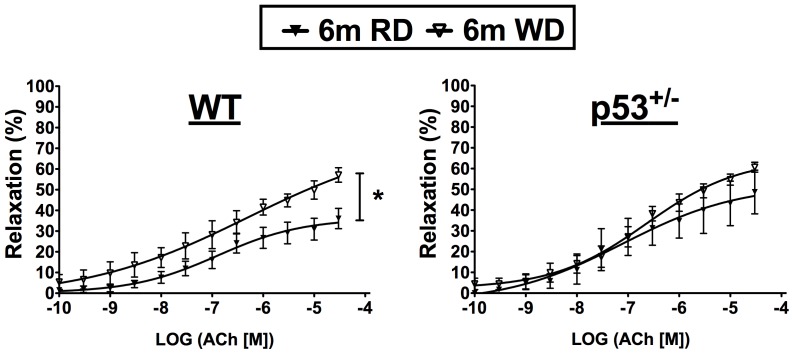
Adaptive endothelium-dependent mechanisms are not activated in p53^+/^^−^ mice. Effect of WD on non-NO/non-prostacyclin endothelium-dependent relaxation of aorta from WT and p53^+/−^ mice. Endothelium-dependent relaxation by acetylcholine (Ach) of aortic segments from WT and p53^+/−^ mice was assessed in the presence of NOS inhibition (L-NNA, 100 μM) and nonspecific COX1/2 inhibition (indomethacin, 10 μM). Data are means±SEM. n = 5–10.

After 3 months on WD, maximal relaxation induced by Ach in WT mice increased to match that evoked in p53^+/−^ mice (Table 4, Fig. 6), demonstrating that the vascular endothelium of healthy mice exposed to deleterious diets can adapt, at least initially, to stressful environments. VSMC function was not affected by WD, as evidenced by the similar relaxing effect of SNP and the maximal contraction evoked by 127 mM KPSS in WT and p53^+/−^ mice (data not included). Indomethacin did not further increase the relaxation induced by Ach in WT mice (Table 4). While no difference in relaxation were observed after blockade of the NO pathway with L-NNA alone (data not included), L-NNA and indomethacin combination revealed the presence of a more effective non-NO/non-prostacyclin (PGI_2_)-relaxing pathway in WT mice fed WD compared to RD (Table 4, Fig. 8). In p53^+/−^ mice, similar endothelium-dependent relaxation was seen in the presence of L-NNA and indomethacin in mice fed either diet (Table 4, Fig. 8), indicating that this non-NO/non-PGI_2_ pathway is not further activated by WD in p53^+/−^ mice.

### Oxidative stress and SOD2 activity

**Figure 9:**
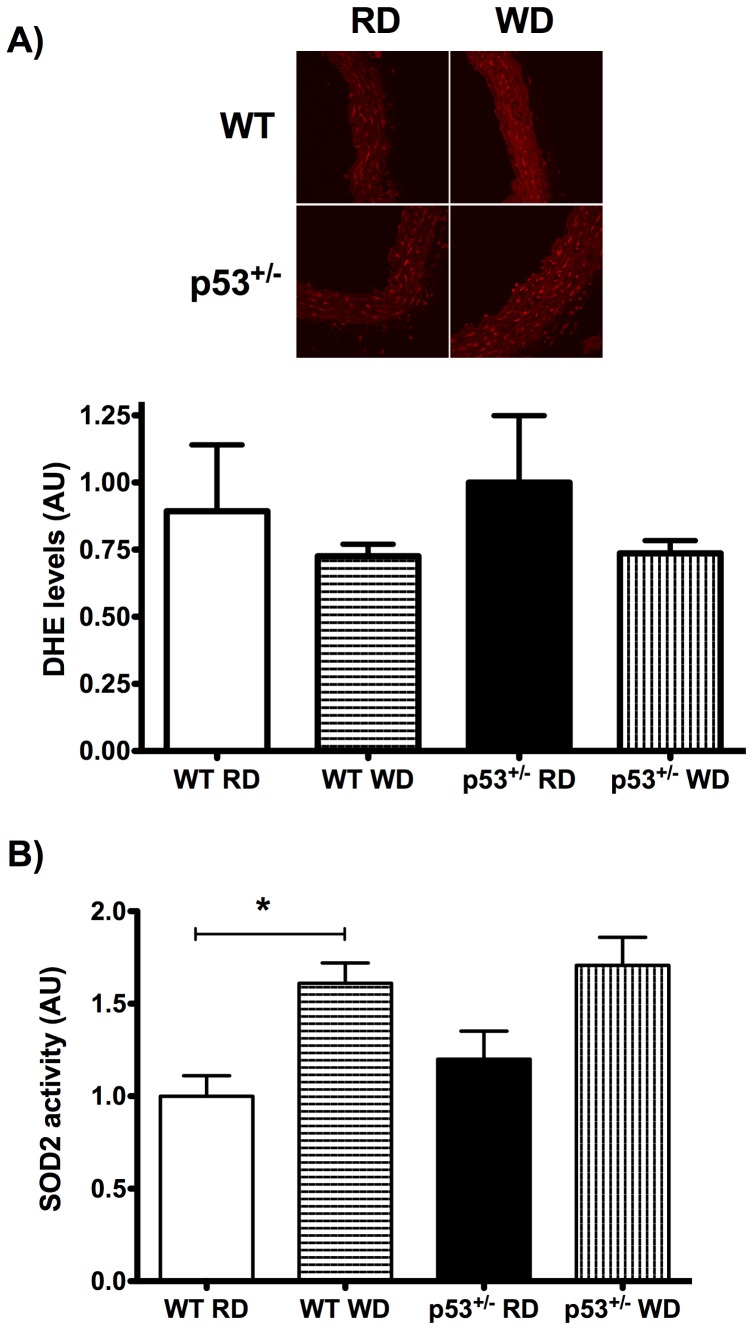
Aortic oxidative stress level and SOD2 activity. (A) Staining with dihydroethidium (DHE, red) of WT and p53^+/−^ 6-month old mice fed a regular diet (RD) or a 3-month western diet (WD). Data are mean±SEM; *n  = * 3–4. (B) Effect of the WD on superoxide dismutase 2 (SOD2) activity in aortic segments from WT and p53^+/−^ mice. Data are mean±SEM; *n  = * 3–8. *p<0.05 compared to RD with matching genotype.

Oxidative stress limits endothelial function [18]. We observed similar levels of global oxidative stress in aorta from all groups (Fig. 9A). Importantly, however, the WD augmented SOD2 activity in WT mice (Fig. 9B) further supporting the ability of young WT mice to up-regulate antioxidant enzyme activity in response to the stress created by the WD. In p53^+/−^ mice, although not statistically significant (p = 0.08), SOD2 activity tended to increase as well following the WD (Fig 9B). Consequently, SOD2 activity was similar in WT and p53^+/−^ mice fed a WD (Figure 9B).

## Discussion

The two major findings of our study are that between 3 and 6 months of age, the endothelial dilatory function was maintained in mice expressing low p53 levels, while it degraded in the control strain, suggesting that a lower p53 activity confers endothelial resistance to age-related stress. Second, the WD did not elevate circulating levels of cholesterol in p53^+/−^ mice unlike in the control strain. This effect was associated with a low-grade inflammatory response if any, a lack of change in vascular endothelial function and, thus, absent expression of adaptive and compensatory dilatory mechanisms in response to the combination of aging and metabolic stress. Our data suggest that, independently of its direct endothelial effects and by accelerating cholesterol elimination, the low activity of the p53 pathway permitted altogether the expression of a phenotype that is protective of the cardiovascular system.

Complete abrogation of p53 expression in mice (p53^−/−^) leads to the development of carcinomas as early as age 10 weeks, causing their premature death [17], [31]. In p53^+/−^ mice, however, carcinomas occur later in life, starting at 9 months of age (∼2% of mice) [31], [32]. There is therefore no doubt that impaired expression of p53 and its subsequently poor increase in expression in response to stress are deleterious by not regulating the cell cycle following damage, and thus, not promoting growth arrest and senescence or apoptosis [17], [33], [34]. The role of p53 is, however, not limited to tumour suppression. Atherosclerotic plaque progression is accelerated in p53^−/−^ mice fed a WD and in severely dyslipidemic mice [35]–[37], demonstrating that preventing the elimination of damaged cells elicits not only tumours but also premature atheromas, among other pathologies. We previously reported that activity of the p53/p21 pathway was heightened in EC isolated from atherosclerotic patients [12], and even more so in EC from active smokers [13], as seen in advanced atherosclerotic plaques [7]. On the other hand, constitutive p53 hyperactivity evokes premature death (23% reduction of median lifespan) in mice due to oversensitivity to damage and accelerated reparative cell turnover [38]. It has been proposed that p53 activation induces free radical-dependent irreversible damage and cell death by blunting the expression of antioxidant enzymes [39]. In contrast to the response to damage associated with augmented p53 activity, it has been suggested that low p53 levels are protective because they stimulate antioxidant defense mechanisms [10]. We observed that reduction of free radical generation with an antioxidant was essential to delay p53-associated senescence of EC isolated from arteries of atherosclerotic patients [14]. It appears that p53 and reactive oxygen species (ROS) participate in a complex crosstalk where p53 can favour either anti- or pro-oxidant pathways to control cell fate [6]. Recent data indicate that low p53, despite its probable low capacity to induce a protective DNA damage response could promote cellular protection [10] but only for a limited time [17], [31].

We observed that endothelial function was better maintained with time in aorta isolated from p53^+/−^ mice than in WT mice (Table 4, Fig. 6), suggesting that the p53^+/−^ genotype is associated with a better resistance to age-related stress. In support of this suggestion, the age-dependent decline in endothelial function of arteries isolated from WT mice could be reversed by either nonspecific COX1/2 inhibition or by enhancing cellular antioxidant capacities with cell-permeable PEG-catalase (Table 4). We reported previously that a similar age-associated (from 3 to 6 months) decline in dilation of renal arteries in C57Bl/6 mice was attributed to heightened TXA_2_ release and oxidative stress; the former was prevented by antioxidant treatment with the polyphenol catechin [18]. While TXA_2_ does not seem to contribute to the reduction of maximal relaxation in 6-month-old WT mice (Fig. 7), COX1/2 can produce either prostanoids or ROS that have been shown to be pro-constrictors [40]. For example, PGF_2α_ mediates constriction in aorta from hamsters [41], and COX-derived ROS constrict the aorta of spontaneously hypertensive rats [42]. In aged rats, an increase in endothelium-derived constricting factor produced by both COX isoforms is responsible for endothelial dysfunction of the femoral arteries [43]. It appears therefore, that low p53 expression mimics the protective effect of catechin in WT mice [18], presumably by reducing COX-derived constricting factors.

A WD has been shown to induce atherosclerosis in aged LDLR^−/−^ mice because they were unable to up-regulate the expression of antioxidant enzymes [44]. In WT mice, however, our data demonstrate that a hypercholesterolemic WD engenders stress as evidenced by a rise in circulating pro-inflammatory KC (Table 3). KC, the IL-8 mouse ortholog in humans, is responsible for monocyte arrest in early atherosclerotic lesions [45], [46], contributing to the development of inflammation and atherosclerosis [47]. This stress is, however, counteracted by a compensatory increase of SOD2 activity (Fig. 9B) and the expression of a higher non-NO/non-PGI_2_ pathway (Table 4, Fig. 8). SOD up-regulation likely contributes to maximal endothelium-dependent relaxation by Ach, as reported previously [21], [48]. Although these protective compensatory adaptations to their new environment are likely temporary, they concur with previous results from our group and others [49]–[51]. Hyperactivity of the endothelium-derived hyperpolarizing pathway in young dyslipidemic mice [50], increased NO production in hypercholesterolemic rabbits [51] and enhanced aortic relaxation due to increased leptin in mice fed high-fat diet [49] were reported. Therefore, the rise in circulating cholesterol, known to induce inflammation, oxidative stress and atherosclerosis in humans [16], [52], elicits a transitory adaptive defense response in the vascular endothelium. This adaptive response, however, may not be possible in old mice as shown by others in 12-month old LDLR^−/−^ mice [43].

In young p53^+/−^ mice, however, the WD did not evoke hypercholesterolemia, only increasing glycaemia and body weight; furthermore, circulating KC did not rise significantly and endothelial function was not altered with age. Therefore, the rise in blood glucose in the absence of hypercholesterolemia was not a significant stress stimulus in these mice. Nonetheless, SOD2 activity tended to increase and was not different from that measured in the aorta of WT mice suggesting that p53^+/−^ mice are still capable of sensing a change in the metabolic environment and adapt to this change. Therefore, while the low expression of p53 likely explains the lack of hypercholesterolemia in these mice, the role of p53 in the vascular adaptive response remains questionable.

It has been shown that lowering circulating cholesterol levels ameliorates endothelial function [53], [54]; the absence of hypercholesterolemia when fed a WD could not alter endothelial function in p53^+/−^ mice. Blood glucose increased similarly in both groups of mice, while triglycerides slightly decreased as previously reported by others [55], [56]. In these young mice, the rise (∼ 35%) in glucose alone is therefore not sufficient to induce a significant endothelial stress. The question is why the WD did not raise cholesterol? The expression of hepatic HMG CoA reductase, LDLR, apoB and PCSK9 (Fig. 2) was not different from values measured in WT mice, suggesting similar cholesterol synthesis and LDL uptake. We found, however, that bile acids were elevated in the plasma of p53^+/−^ mice (Table 3). Bile acid metabolism is the main cholesterol clearance pathway. Resins are lipid-lowering drugs that can reduce LDL-cholesterol by approximately 20%, by binding bile acids in the intestine and leading to its excretion in the feces [57]. By sequestering bile acids and lowering their amount, resins force the liver to produce more bile acids with cholesterol as substrate, thereby lessening available cholesterol in the blood [57]. Because excess bile acids can culminate in damage [58], their metabolism is tightly regulated (Fig. 3A). p53 has recently been implicated in the regulation of bile acid metabolism [26], [27]. Together with the transcription co-activator mixed-lineage leukemia 3 (MLL3), p53 can bind the SHP promoter to stimulate its expression [26]. p53 activation with doxorubicin in p53^−/−^, MLL3^−/−^ and SHP^−/−^ mice did not increase bile acid concentration in the serum and liver in contrast to WT mice [26], [27]. In line with this finding, we noted that SHP expression was lower in the liver of p53^+/−^ mice, whereas Cyp7A1 mRNA, the enzyme responsible for bile acid production, was higher (Table 3, Fig. 2). However, despite an increase in gene expressions of p21, p53 and SHP, DOX did not lower Cyp7A1 expression in HepG2 cells (Fig. 5), contrasting with the report by Kim and colleagues [26] performed in the same experimental conditions. The reason for this discrepancy is unknown. The response to DOX of the HepG2 cells, in which p53 activity is constitutively increased, may be biased and not representative of the physiological regulatory role of p53 *in vivo*. It is a limitation of this study because cultured cells are under stress and have elevated levels of p53, which is likely accompanied by numerous intracellular adaptations that may blunt responses otherwise expected *in vivo*. Finally, we did not clearly identify the precise lipoprotein fraction responsible for the rise in plasma cholesterol in the WT mice, which was not an objective of our study. Direct measures of LDL-cholesterol, however, showed that it did not change, suggesting that non-HDL-cholesterol (possibly VLDL) may be implicated in the hypercholesterolemia observed in WT mice following the WD. This has been previously reported to occur in mice fed a high fat diet [59]. Nonetheless, and on aggregate, these data indicate that enhanced bile acid synthesis from cholesterol in p53^+/−^ mice blunted the hypercholesterolemia normally associated with a WD; this, consequently, could not stress the vascular endothelium. The latter explanation is however speculative and represents the main limitation of the study. Our study was not able to demonstrate a clear link between the endothelial function and p53 genotype. This would have been possible if the WD had induced a similar metabolic profile in the two mouse groups, the initial purpose of the study. Nonetheless, the vascular endothelium was resistant to the deterioration of its function with aging that was observed in WT mice (Table 4).

In conclusion, our data demonstrate that low p53 activity is coupled with constitutively faster cholesterol turnover from the diet. The elevated levels of bile acids in p53^+/−^ mice, however, may contribute to heightened risk of premature carcinogenesis in later life. In addition, our data suggest that p53 regulate endothelial function and confer age-related stress resistance. However, p53 may not be involved in the adaptation to stress of the endothelium since SOD2 activity increased in both mouse strains when challenged with a WD. Furthermore, the fact that a high-fat diet did not increase cholesterol levels in the p53 mutant mice is the direct demonstration of the ubiquitous functions of p53.
